# Bright Light During Wakefulness Improves Sleep Quality in Healthy Men: A Forced Desynchrony Study Under Dim and Bright Light (III)

**DOI:** 10.1177/07487304221096910

**Published:** 2022-06-22

**Authors:** R. Lok, T. Woelders, M. C. M. Gordijn, M. J. van Koningsveld, K. Oberman, S. G. Fuhler, D. G. M. Beersma, R. A. Hut

**Affiliations:** * Chronobiology Unit, Groningen Institute for Evolutionary Life Sciences, University of Groningen, Groningen, the Netherlands; † University of Groningen, Leeuwarden, the Netherlands; ‡ Department of Psychiatry and Behavioral Sciences, Stanford University, Palo Alto, California, USA; § Chrono@Work B.V., Groningen, the Netherlands

**Keywords:** light, sleep-wake dependent sleep pressure variation, circadian variation, forced desynchrony, polysomnography, NREM sleep, REM sleep, subjective sleep quality

## Abstract

Under real-life conditions, increased light exposure during wakefulness seems associated with improved sleep quality, quantified as reduced time awake during bed time, increased time spent in non-rapid eye movement (NREM) sleep, or increased power of the electroencephalogram delta band (0.5-4 Hz). The causality of these important relationships and their dependency on circadian phase and/or time awake has not been studied in depth. To disentangle possible circadian and homeostatic interactions, we employed a forced desynchrony protocol under dim light (6 lux) and under bright light (1300 lux) during wakefulness. Our protocol consisted of a fast cycling sleep-wake schedule (13 h wakefulness—5 h sleep; 4 cycles), followed by 3 h recovery sleep in a within-subject cross-over design. Individuals (8 men) were equipped with 10 polysomnography electrodes. Subjective sleep quality was measured immediately after wakening with a questionnaire. Results indicated that circadian variation in delta power was only detected under dim light. Circadian variation in time in rapid eye movement (REM) sleep and wakefulness were uninfluenced by light. Prior light exposure increased accumulation of delta power and time in NREM sleep, while it decreased wakefulness, especially during the circadian wake phase (biological day). Subjective sleep quality scores showed that participants rated their sleep quality better after bright light exposure while sleeping when the circadian system promoted wakefulness. These results suggest that high environmental light intensity either increases sleep pressure buildup during wakefulness or prevents the occurrence of micro-sleep, leading to improved quality of subsequent sleep.

Humans spend nearly one-third of their lives sleeping (Callaway, 1988). While asleep, individuals do not eat, drink, or reproduce and are vulnerable due to reduced responsiveness to environmental stimuli. Although there is no general consensus on the fundamental function of sleep, possible explanations include saving calories, enhancing the immune system, increasing brain connectivity, maintaining brain function, and restoring memory and mental and physical functions (reviewed in Krueger et al., 2016). In rodents, lack of sleep leads to excessive body weight loss, skin lesions, and ultimately death (Rechtschaffen et al., 1989), indicating a crucial role for sleep. Reduced sleep duration and quality (i.e., increased sleep onset latency, increased amount of intermittent wakefulness, and reduced sleep intensity) have detrimental consequences in humans, including impaired physical and mental health, reduced feelings of vitality and performance (Belenky et al., 2003; Van Dongen et al., 2003, 2004), and learning and memory problems (Saygin et al., 2017). In the Netherlands alone, 3.3 million (25.4%) women and 2.6 million (19.6%) men suffer from impaired sleep quality at night (Tiemeier and van Someren, 2017).

The two-process model of sleep regulation suggests that sleep is regulated by a homeostatic and a circadian factor (Borbély et al., 2016; Daan et al., 1984). The homeostatic factor represents sleep pressure, which increases with prolonged time awake and dissipates during sleep. The circadian process is a reflection of the circadian system, synchronized by the circadian pacemaker (suprachiasmatic nucleus [SCN]), which alternates between periods with high- and low-sleep propensity independent of prior sleep and wake periods (Borbély, 1982; Borbély and Achermann, 1999). Complex interplays between SCN activity and its effects on the ventrolateral preoptic nucleus (VLPO; Gaus et al., 2002; Sherin et al., 1998; Szymusiak et al., 1998), sub-paraventricular zone (SPZ; Lu et al., 2001), dorsal medial hypothalamus (DMH; Chou et al., 2003; Deurveilher and Semba, 2005; Lu et al., 2001), and lateral hypothalamus (LH; Saper et al., 2001) are thought to regulate timing of sleep During sleep, the mammalian brain alternates between rapid eye movement (REM) and non-rapid eye movement (NREM) sleep (Scammell et al., 2017; Weber and Dan, 2016). The switch between these states occurs in an ultradian rhythm of approximately 90 min.

Sleep quality can be measured with polysomnography (PSG), in which higher sleep quality is reflected by shorter sleep onset latencies, fewer awakenings, higher amounts of NREM sleep, and more intense NREM sleep (measured as power in the delta band; Ibáñez et al., 2018). Several studies report elevated amounts and intensities of NREM sleep in response to increased daytime daylight exposure (Wams et al., 2017) or decreased sleep quality after diminished artificial light exposure during the daytime (Korf et al., 2017). These effects are defined as acute effects of light exposure. Light is known to elicit both image and non-image-forming (NIF) responses (Pittendrigh and Daan, 1976). Image-forming responses predominantly occur through classical photoreceptors, such as rods and cones, with projections to the visual cortex (Horvath et al., 1999), while NIF effects are mainly transmitted through intrinsically photosensitive retinal ganglion cells (ipRGCs; Provencio et al., 1998, 2000). These cells project to hypothalamic regions including the SCN (the master pacemaker in the brain) via the retinohypothalamic tract (Hattar et al., 2002). NIF effects of light have proven essential for entrainment of the circadian system (Cajochen et al., 2005, 2011; Geerdink et al., 2016), which can affect sleep quality (Grønli et al., 2016), defined as chronic effects of light exposure. For example, light exposure in the (late) evening decreases sleep quality (Cajochen et al., 1992; Dijk et al., 1991) since it stimulates the phase delay portion of the human phase response curve (Khalsa et al., 2003; Papantoniou et al., 2014). This effect of light on sleep mediated by the circadian system, however, does not exclude a possible direct effect of light on subsequent sleep independent of the circadian system (Cajochen et al., 2019; Horne and Walmsley, 1976; Wams et al., 2017). Our hypothesis therefore is that light effects on sleep occur independent of circadian clock phase alterations. To accurately assess this, we conducted a forced desynchrony (FD) experiment where bright light (BL) and dim light (DL) effects on sleep were studied at multiple clock phases, without altering the phase angle of entrainment of the electroencephalogram.

## Materials and Methods

For an elaborate version of the “Materials and Methods” section, including participant information and prove-of-principle of the BL FD conditions (in which cortisol data indicate no evidence for non-uniform phase progression in the BL FD), please see Lok et al., (2022b).

### Participant Information

Participants were healthy (n=8), non-sleep deprived males with a regular sleep-wake cycle (as assessed by 2-week actigraphy proceeding the in-lab part of this study) and without sleep problems (as assessed by the Pittsburgh Sleep Quality Index), between the ages of 20 and 30 years (average ± Standard Error of the Mean (SEM); 24.0 ± 1.16). All participants provided written informed consent and received financial compensation for participation. The study protocol, screening questionnaires, and consent forms were approved by the medical ethics committee of the University Medical Center Groningen (NL54128.042) and were in agreement with the Declaration of Helsinki (2001).

### Protocol

**Figure 1.**
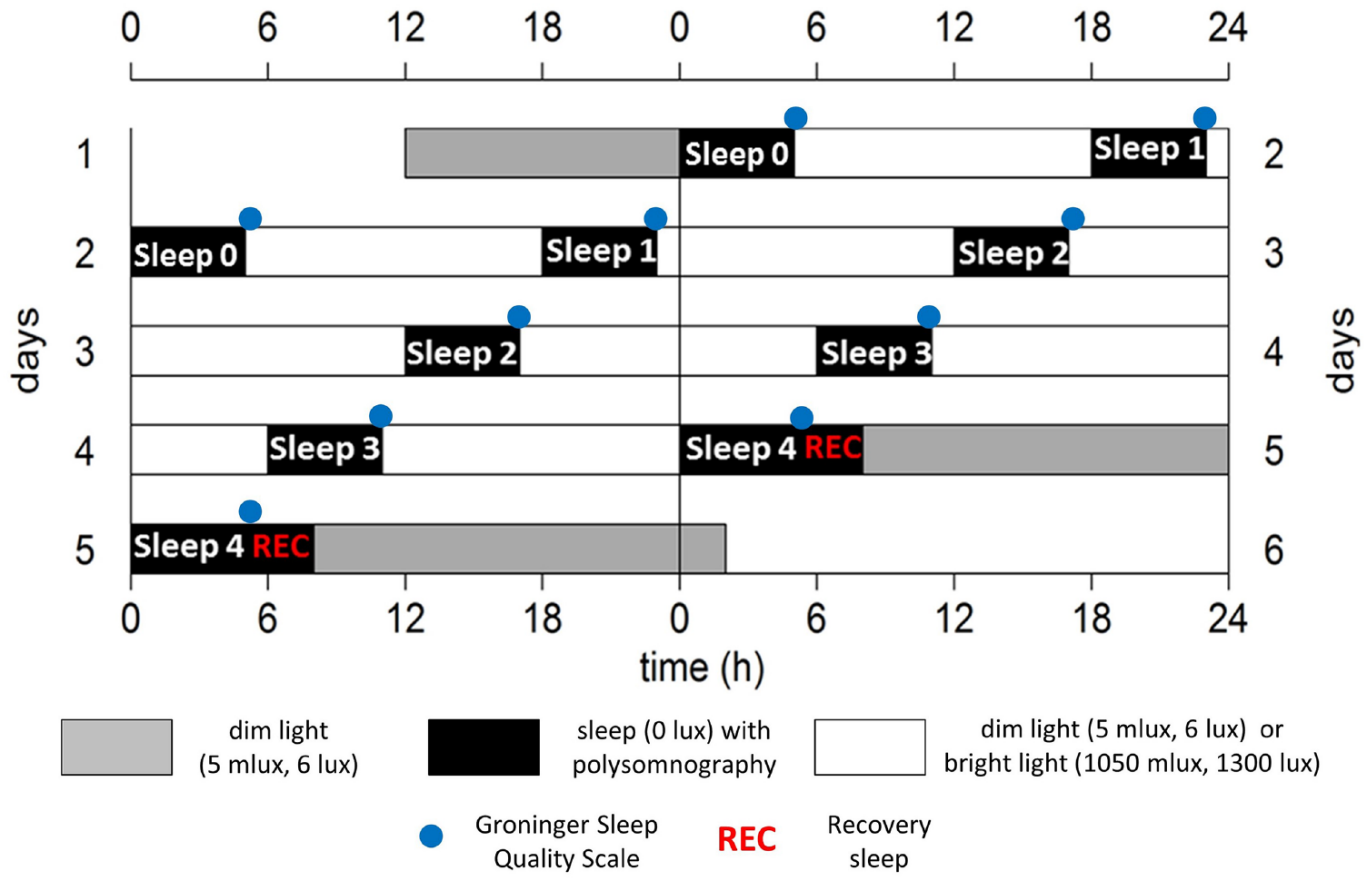
Schematic representation of the experiment design for an individual with a HSon at 0000 h, double-plotted. Gray bars indicate dim light (6 lux) conditions preceding and following the forced desynchrony protocol. Black bars represent intervals for sleep (5 h) with polysomnography, while lights are off (0 lux). Scheduled sleep episodes are labeled for optimal clarity ranging from 0 to 4, followed by a 3-h interval for recovery sleep (REC). Blue dots indicate timing of completing the Groninger Sleep Quality Scale. White areas represent wakefulness in either polychromatic white dim light (6 lux, 5 melanopic lux) or bright light (1300 lux, 1050 melanopic lux) conditions. The protocol lasted for 72 h, therefore comprising a full beat between the imposed and internal cycles (3 × 24 h = 72 h, 4 × 18 h = 72 h).

Participants arrived at the human isolation facility of the University of Groningen 10 h before habitual sleep onset (HSon; assessed with the Munich Chronotype Questionnaire [MCTQ], Roenneberg et al., 2003, and confirmed by actigraphy). Upon arrival, individuals were equipped with 6 scalp electrodes to measure PSG (O3, O4, C3, C4, F3, and F4), 2 electro-oculogram (EOG) electrodes, 2 electromyography (EMG) electrodes underneath the chin, and reference electrodes placed on the left and right mastoid. The FD protocol commenced with scheduled sleep in darkness, starting at HSon (Figure 1). After 5 h of darkness (sleep 0, habituation night), participants were woken up under polychromatic white light of either DL (6 lux) or BL intensity (1300 lux); both intensities measured vertically at the level of the eye (for more specifications of the light, see paragraph below). Immediately after awakening, the Groninger Sleep Quality Scale (GSQS; Leppämäki et al., 2003) was completed to assess subjective sleep quality. Participants remained awake under these respective DL or BL conditions for 13 h, during which they were allowed to read, study, or watch movies. Physical activity (at any intensity) or naps were not allowed. After 13 h of wakefulness, participants were instructed to go to bed, and light intensities were set to 0 lux (sleep 1). The length of the FD paradigm should cover an integer number of beats of the two involved rhythms. This is required for the mathematical disentanglement of sleep-pressure-related variation from circadian variation. Here, the 18-h FD cycle, consisting of 5 h for sleep and 13 h for wakefulness, was repeated 4 times, resulting in a 72-h FD protocol (4 times 18 h matches 3 times 24 h). We chose a short FD design (5 h of sleep – 13 h of wakefulness) to minimize the duration of the FD protocol, since BL exposure can influence the internal period, therefore confounding comparisons within individuals. According to simulations with the two-process model of sleep regulation, sleep pressure levels did not systematically increase or decrease over the course of the FD protocol (Lok et al., 2022b, Suppl. Fig. S2), nor did sleep pressure change internal period substantially. Further simulations indicated that 5 h of sleep and 13 h of wakefulness minimized chances of non-uniform phase progression, since with this ratio, light exposure is timed in such a way that both the delay and advance zone of the phase response curve are hit equally (Lok et al., 2022b, Suppl. Fig. S1). After these 4 cycles, a short obligatory awakening was scheduled during the last sleep period to fill in the GSQS, and participants were offered an additional recovery sleep opportunity. To avoid possible effects of sleeping outside the normal sleep phase on subsequent Dim Light Melatonin Onset (DLMO) assessment, all participants were woken up after 3 more hours. Participants were assigned to the DL or BL in a counterbalanced manner (*n* = 4 starting with DL). After completion of the first FD protocol, study volunteers were allowed to go home and returned after at least 3 weeks to participate in the second FD protocol under the other light condition. The experiment was conducted between February and May 2018.

### Light Exposure

Polychromatic white DL (6 lux, 5 melanopic lux) and BL (1300 lux, 1050 melanopic lux) was provided via ceiling-mounted Philips fluorescent light tubes (see Lok et al., 2022b, Suppl. Table S1 and Fig. S3 for all α-opic illuminance values and spectral composition). Light intensity was measured on the vertical plane at the level of the eye. Each participant was exposed to the same light intensity throughout the light phase of one FD protocol.

### Data Preprocessing: PSG

Data were collected using the TEMEC EEG (Vitaport, 28 channels, TEMEC instruments BV, Kerkrade, the Netherlands) system. Electrode impedance was maintained below 5 kΩ. All PSG signals were analyzed using Vitascore (V1.60, TEMEC instruments BV, Kerkrade, the Netherlands) and all EEG, EMG and EOG signals were referenced to the contralateral mastoid reference signal. After artifact removal, recordings were scored manually according to the criteria of Rechtschaffen and Kales (1968) with a 50-Hz notch filter, and high-pass (0.3 Hz) and low-pass (32 Hz) filter settings applied. Sleep onset was determined as the time of the start of at least 5 consolidated minutes (not interrupted by wake episodes) of sleep (stage N1, N2, N3, or REM) (Hori et al., 2001). Power spectra were computed per 4-sec epoch using tapered Fast Fourier Transform (Vitascore, 30 sec. reporting epoch). NREM power density was calculated per epoch in the delta band (0.5-4 Hz). To ensure accurate assessment of sleep-wake-dependent sleep pressure variation (see below), successful PSG recordings of a participant have to be present at all 4 sleep opportunities. Recordings failed for one participant at sleep opportunity 2, while sleep recordings failed for another participants at sleep opportunity 4, resulting in *n* = 6 participants. Original data are depicted in Supplementary Figures S1-S4, and Table S1. Sleep 0 (Figure 1) was used as habituation night and therefore not included in any statistical analysis. Due to technical failures, recovery sleep could only be analyzed in 4 individuals.

### Data Preprocessing: Subjective Sleep Quality

GSQS scores range from 0 to 14, with higher scores indicating lower subjective quality of sleep (Leppämäki et al., 2003). Data collected during BL exposure were expressed relative to subjective sleep quality at that same scheduled sleep in DL. A higher score therefore indicates better subjective sleep quality. One subject did not correctly complete the questionnaire and was therefore excluded from subjective sleep quality analyses (*n* = 7).

### Sleep-Wake Dependent Sleep Pressure Variation (Process S)

To assess effects of light exposure on subsequent sleep, only scheduled sleep episodes 1 to 4 were included in the analysis. Time in each sleep stage was expressed relative to total sleep time and divided into 10- and 100-min bins (Suppl. Fig. S5). Sleep-duration-related variation was calculated by plotting all data as a function of time since sleep onset and averaging those per time point (process S). Data were fitted by a Locally Weighted Least Squares Regression “loess fit” in RStudio (version 1.0.136).

### Circadian Variation (Process C)

To determine circadian variation, original data were calculated as a function of circadian phase, defined as the timing of DLMO before the start of the protocol subtracted from time in FD, and divided by tau. These values were divided into 30-degree bins.

### Statistics

RStudio (version 1.0.136) was used for statistics and graphics. Generalized linear mixed models (package “lme4”) were constructed with light condition as independent variable, time since sleep onset, and circadian phase as a fixed effect (categorical variable), and added interaction terms between time since sleep onset and circadian phase, time since sleep onset and light, and circadian phase and light condition. Subject ID and visit were included as random effects to control for between-subject variation and possible order effects. Critical 2-sided significance level alpha was 0.05 for all statistical tests. To further investigate the interaction between circadian variation and light condition on delta power accumulation, time spent in NREM, REM, and wakefulness, a sine wave with formula $y=c+a\cdot \sin\left(\frac{2\cdot \pi \cdot (x+b)}{360}\right)$ , with *c* representing the constant, *a* the amplitude, *x* the time (in degrees), and *b* the phase, was fitted to the data per light condition. The “summary” function was used to obtain statistics, and a significant amplitude is considered and reported as a significant circadian modulation. Significant interaction terms (*p* < 0.05) between light and time effects allowed for calculations of significant light effects by contrast analyses for all combinations of circadian time and time since sleep onset (corrected for multiple testing [Tuckey correction], package “lsmeans”). To ensure sufficient sample size (*n* ≥ 4) for each combination of “time since sleep onset” and “circadian clock phase,” we constructed a separate linear mixed model to calculate significance of the interaction terms of these variables with light condition. Contrast analyses [Tuckey correction], were conducted on all combinations of circadian time and time since sleep offset. Contrasts were constructed in 90-degree bins, with sleep-dependent changes in bins of 50 min. Significant contrasts are depicted in 3-dimensional graphs, in which circadian variation is represented on the *x*-axis, wake-duration-related variation on the *y*-axis (in 50 minute bins) and BL scores subtracted from DL scores on the z-axis (with colors indicating the direction and magnitude of statistically significant light effects). Combinations of wake-duration-related variation and circadian variation that contain data of fewer than 4 individuals are considered underpowered and presented as “missing data” in gray. To estimate light effects during the time course over the projected night course, sleep was predicted to start 2 h after DLMO (Burgess et al., 2003; coinciding with circadian phase 30) and last for 8 h (until circadian phase 150), after which 13 h of wakefulness commenced. Overall effects are indicated in tables. Data are presented as average ± SEM, unless indicated otherwise.

### Sleep Accumulation

Accumulation of different sleep parameters was determined by integration of data over time. To determine effects of light on accumulated data, general linear hypothesis testing (package “Multcomp”), including simultaneous tests and confidence intervals for general linear hypotheses, was used. Critical 2-sided significance level alpha was kept at 0.05 for all statistical tests. Overall effects are indicated in the text. Significant interaction terms (*p* < 0.05) between light and time effects allowed us to calculate significant light effects by contrast analyses, as indicated in each graph by an asterisk.

## Results

### Light Effects on Sleep Architecture

#### Sleep-Wake Dependent Sleep Pressure (Process S)

**Figure 2.**
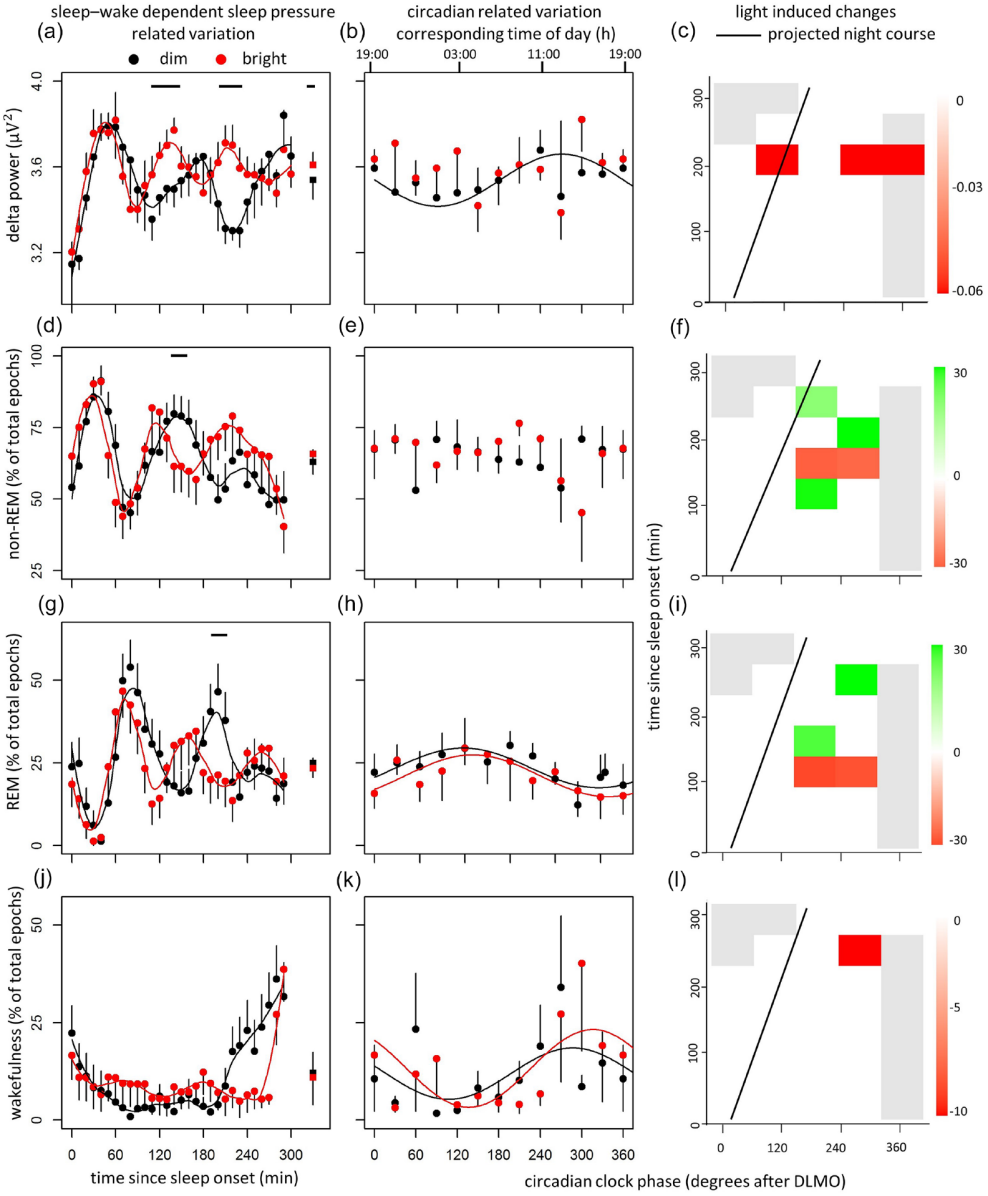
Data of delta power (top panel); time in NREM and REM (middle panels), and wakefulness (bottom panel). Time course of delta power (a), time spent in NREM sleep (e), REM sleep (g), and wakefulness (j) during the FD protocol plotted as time since sleep onset. Data replotted as circadian phase in degrees after Dim Light Melatonin Onset (DLMO) (b, e, h, and k), for delta power, time spent in NREM sleep, REM sleep, and wakefulness, respectively, with corresponding time of day (h) depicted on the top axis. Contrast analysis describing light-induced decreases for all combinations of circadian clock phase and time since sleep onset for delta power (d), time in NREM sleep (f), time in REM sleep (i), and wakefulness (l). Data represent mean ± standard error of the mean, with 6 subjects per group. Black dots indicate data collected in dim light, red dots represent data collected in bright light, and black and red squares represent averages over all data points under dim light and bright light, respectively. Gray line indicates the projected time course over a regular night. Significant differences between light conditions (*p* < 0.05) are indicated by horizontal black bars (a, d, g, j) or colored rectangles (c, f, i, l). Gray rectangles indicate combinations of wake-duration-related variation and circadian clock phase containing data of less than 4 individuals (c, f, i, l). Abbreviations: NREM = non-rapid eye movement; REM = rapid eye movement; FD =forced desynchrony.

Both time since sleep onset and light exposure significantly affected delta power, indicating more delta power following BL exposure (Figure 2a, Table 1). Although there were significant effects of time since sleep onset on time in NREM sleep, REM sleep, and wakefulness, these parameters were not significantly altered by light exposure (Figure 2c, 2e, and 2g, Table 1).

**Table 1.** Summary of statistics of sleep-wake dependent sleep pressure variation (process S); circadian variation (process C); interaction between process S and light, and process C and light; and additive effects of bright light exposure (defined as a light effect independent of process S and/or C).

	Sleep-Wake Dependent Sleep Pressure Variation (Process S)		Circadian Variation (Process C)		Interaction (Process S x Light)		Interaction (Process C x Light)		Additive Effect of Bright Light	
Delta power	*F_(29,882),_ p*	**46.32,** <2.20 x 10^-16^	*F_(11,882),_ p*	**3.28,** 3.61 x 10^-9^	*F_(29,882),_ p*	0.88, >0.05	*F_(11,882),_ p*	0.63, >0.05	*F_(1,882),_ p*	**234.61,** <2.20 x 10^-16^
Non-REM	*F_(29,882),_ p*	**3.45,** 2.67 x 10^-9^	*F_(11,882),_ p*	1.12, >0.05	*F_(29,882),_ p*	0.94, >0.05	*F_(11,882),_ p*	1.38, >0.05	*F_(1,882),_ p*	1.38, >0.05
REM	*F_(29,882),_ p*	**4.09,** 4.38 x 10^-12^	*F_(11,882),_ p*	**2.19,** 0.01	*F_(29,882),_ p*	1.01, >0.05	*F_(11,882),_ p*	0.49, >0.05	*F_(1,882),_ p*	1.04, >0.05
Wakefulness	*F_(29,882),_ p*	**4.30,** 4.59 x 10^-13^	*F_(11,882),_ p*	**11.64,** <2.20 x 10^-16^	*F_(29,882),_ p*	**1.53,** 0.04	*F_(11,882),_ p*	**5.50,** 1.26 x 10^-8^	*F_(1,882),_ p*	0.51, >0.05

Values from linear mixed models on delta power, time spent in non-REM sleep, REM sleep, and wakefulness.

Abbreviation: REM = rapid eye movement.

#### Circadian Sleep Regulation (Process C)

A circadian rhythm in NREM power was assessed under DL (*S*_residuals_ = 0.14, *p* = 0.001), but a non-significant relationship under BL suggests lack of circadian modulation in BL (*S*_residuals_ = 0.27, *p* = 0.09) conditions, showing less NREM power density in the hours following DLMO (Figure 2b, Table 1). There was no circadian contribution on time in NREM sleep (DL; *S*_residuals_ = 14.24, *p* = 0.12, BL; *S*_residuals_ = 17.14, *p* = 0.11; Figure 2d, Table 1). A significant anti-phasic circadian contribution was detected in time in REM sleep (DL: *S*_residuals_ = 11.12, *p* = 0.005; BL: *S*_residuals_ = 11.12, *p* = 0.01; Figure 2f, Table 1) and wakefulness (DL: *S*_residuals_ = 13.1, *p* = 0.002; BL: *S*_residuals_ = 16.2, *p* = 0.001; Figure 2h, Table 1), both independent of light intensity. For alternative bin sizes, see Supplementary Figure S6. For the main effects of process S and process C, see Supplementary Figure S7.

#### Effects During Projected Night Course

Significant interactions were found between circadian phase, time since sleep onset, and light-induced change in delta power, time spent in NREM sleep, REM sleep, and wakefulness, outside the circadian time interval for a regular night (Figure 2c, 2f, 2i, and 2l).

#### Sleep Accumulation

**Figure 3.**
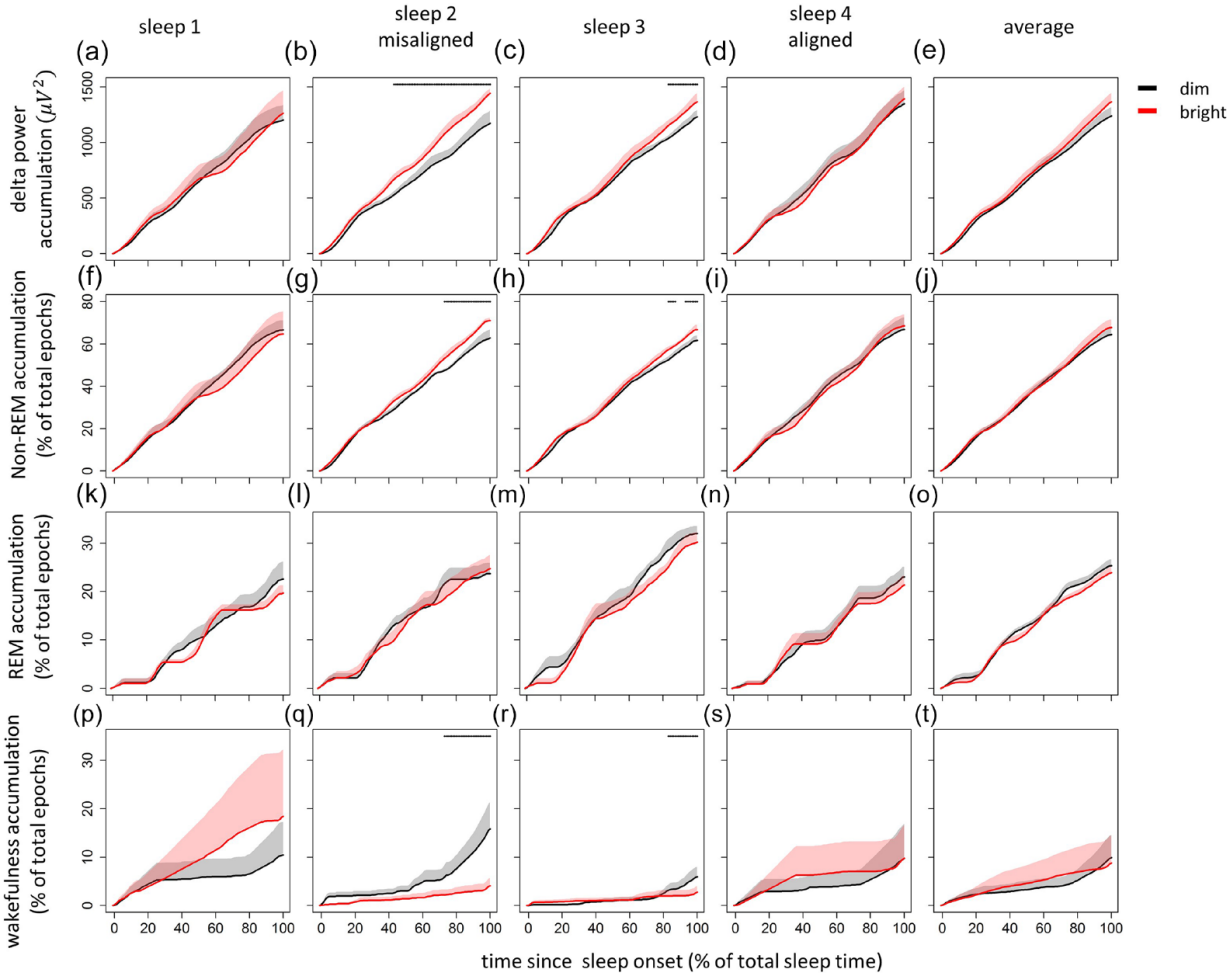
Cumulative amounts of sleep as a percentage of total sleep time, for every scheduled sleep phase and average. Depicted are cumulative amounts of delta power (a-e), time in NREM sleep (f-j), REM sleep (k-o), and wakefulness (p-t). Dim and bright light are depicted in black and red, respectively. Shaded areas indicate standard error of the mean, and horizontal bars in each panel indicate significant differences (α < 0.05). Abbreviations: NREM = non-rapid eye movement; REM = rapid eye movement.

Significant differences between DL and BL conditions were detected in schedule sleep windows 2 and 3, in which significantly more delta power (Figure 3b and 3c) and time in NREM sleep (Figure 3g and 3h) was found after BL exposure, while wakefulness significantly decreased (Figure 3q and 3r). These light effects diverged toward the end of the sleep window. Statistics can be found in Supplementary Table S2. For data on sleep-wake dependent sleep pressure and circadian sleep regulation, please see Supplementary Figures S7 and S8.

## Light Effects on Sleep Quality

### Subjective Sleep Quality

**Figure 4.**
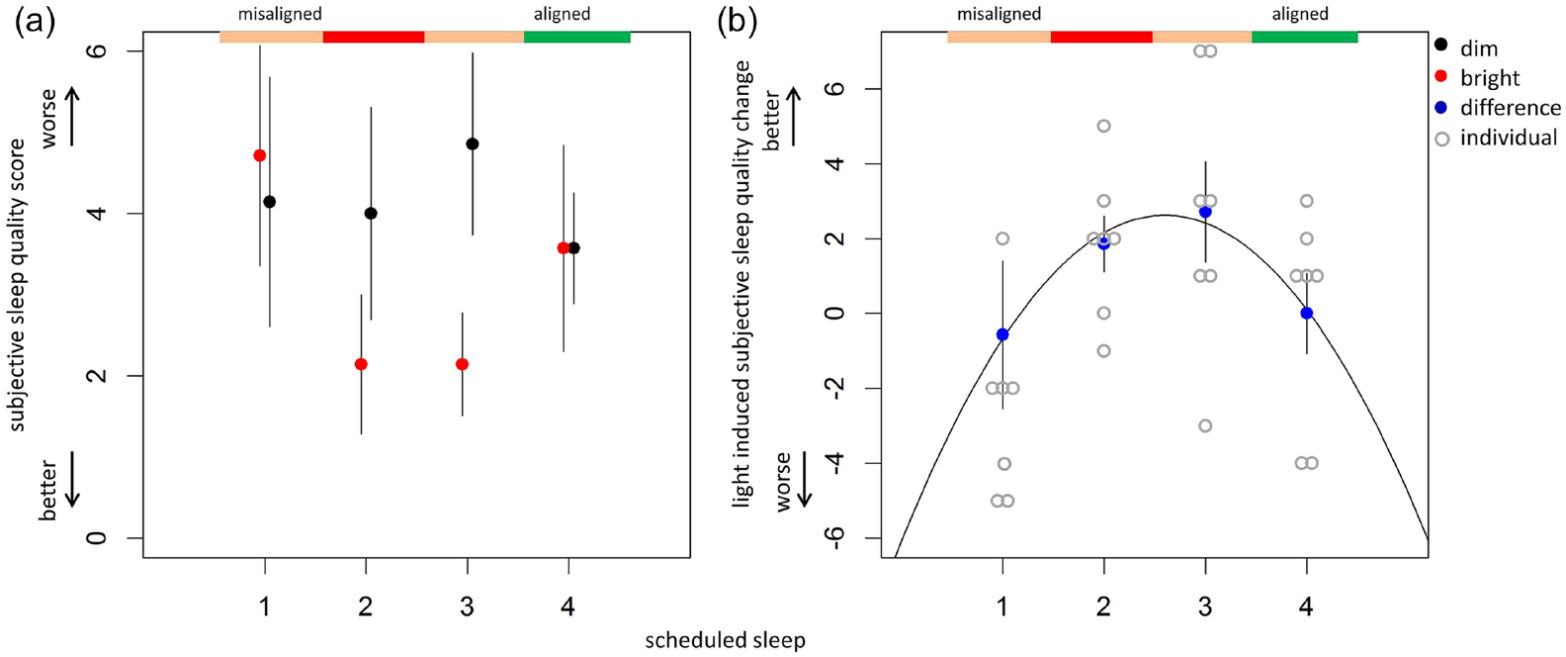
Light effects on subjective sleep quality. Groninger Sleep Quality Scale scores per light conditions (a) and the intra-individual change in subjective sleep quality (b). Subjective sleep quality following bright light exposure was subtracted from subjective sleep quality reported following dim light exposure, where higher scores indicate improved sleep quality after bright light exposure (b).

Within each subject, subjective sleep quality in BL exposure was expressed relative to subjective sleep quality as reported after that same scheduled sleep episode during DL condition (Figure 4b). A quadratic fit $\left(\chi^{2}(1)=5.35,\;p<0.05\right)$ best describes the relation between scheduled sleep and change in subjective sleep quality (black line, Figure 4b), indicating BL improved sleep quality at scheduled sleep 2 and sleep 3, when sleep occurred during the circadian wake phase.

## Discussion

The goal of this experiment was to assess effects of BL exposure on subsequent sleep architecture and quality, independent of circadian clock phase. Results indicate significant modulation in the amount of delta power, time spent in NREM or REM sleep, and wakefulness due to sleep-wake dependent sleep pressure variation (process S). With varying sleep-wake dependent sleep pressure, BL exposure significantly increased delta power. Significant circadian variation (process C) was determined in delta power under DL exposure, but could not be detected following BL exposure, although this might be due to a false negative. There were no significant circadian modulations on time in NREM sleep, while both time in REM sleep and wakefulness showed an anti-phasic circadian modulation, independent of light exposure. Light-dependent improvements in subjective and by PSG assessed sleep quality were found, predominantly when sleep occurred outside the normal sleep phase. Nevertheless, during recovery sleep scheduled during the circadian sleep maintenance zone, light-induced increases in sleep quality as assessed by PSG were also detected.

### Light Effects on Sleep Architecture

The amount of delta power, time spent in NREM, REM sleep, and wakefulness all vary with increasing sleep-wake dependent sleep pressure (process S, Figure 2), as demonstrated by others (Dijk and Czeisler, 1995). Depending on sleep-wake dependent sleep pressure, significant interactions between light exposure and all sleep parameters were probably facilitated by entering different sleep stages at different times between light conditions. Overall, BL exposure significantly increased delta power, without altering any of the other parameters (See also below, section “Light Effects on Sleep Quality”). With varying internal clock time (process C), delta power cycles in phase with wakefulness and in anti-phase with REM sleep (Figure 2), confirming observations by others (Dijk and Czeisler, 1995). In BL however, there was no significant amplitude in circadian rhythmicity in delta power. This suggests a delta power selective effect of light on the internal clock amplitude. Delta power predominantly occurs during NREM sleep, which is regulated in the thalamus and cortex, and results from synchronization of brainwaves (Lancel et al., 1992; Lu et al., 2006; Sapin et al., 2009; Weber et al., 2015). Light effects on internal clock time may therefore be selective for these brain areas, as has been determined in other species (Shuboni et al., 2015). Although the polychromatic white light source used in this experiment stimulates all photoreceptors, it is known that predominantly ipRGCs are responsible for NIF effects of light. A specific subtype of ipRGCs (M1), known to project to thalamic regions, further supports delta selective effects of BL exposure (Schmidt et al., 2007). In addition, direct projections from ipRGCs to the VLPO exist (Hannibal and Fahrenkrug, 2004; Hattar et al., 2006), while lesions of the VLPO are known to decrease amount of NREM and REM sleep (Lu et al., 2000). With varying circadian clock time (process C) time in NREM, REM sleep, and wakefulness did not significantly differ between light conditions; light effects on sleep architecture occur mostly independent of circadian clock time.

Patterns of NREM-REM cycles have been assessed in FD protocols before (Dijk, 1999; Dijk and Czeisler, 1994; Lazar et al., 2015; O’Donnell et al., 2009; Wyatt et al., 1999, 2006), reporting decreased amounts of time in NREM sleep with increasing time asleep, while time in REM sleep and wakefulness increase. Results presented here suggest decreased delta power and time in NREM sleep with progressing time asleep, without significant increases in time in REM sleep (Figure 2). REM sleep particularly occurs at the end of the night, since (1) it negatively correlates with core body temperature minimum (Charles et al., 2017), (2) it is influenced by the circadian drive for wakefulness that increases at the end of the night (Dijk et al., 1987, 1989), and (3) it is controlled by accumulation of REM sleep propensity during NREM sleep (Benington and Heller, 2017). Scheduled sleep episodes in the current paradigm (5 h) may reduce REM sleep occurrence, since the natural latter part of sleep with high REM propensity is skipped.

### Light Effects on Sleep Quality

For each scheduled sleep window, BL exposure also induced increases in both delta power and time spent in NREM sleep, as has been confirmed by others (Korf et al., 2017; Wams et al., 2017). In contrast to literature (Korf et al., 2017; Wams et al., 2017), the light effects assessed here were maximal during the circadian wake phase. Yet, consistent with literature, results presented here did indicate BL-induced improvements in by PSG assessed sleep quality during recovery sleep which was scheduled when the circadian system promoted sleep (Suppl. Fig. S7). Although results assessed during recovery sleep have to be interpreted with caution due to the small sample size, these measurements at least suggest BL effects on subsequent sleep occurring in phase with the circadian clock. Light effects particularly occur at the end of the night, which might be a consequence of the timing of NREM-REM sleep, suggesting a light-induced shift from REM prevalence to NREM occurrence (Wams et al., 2017). This suggests that a sleep opportunity of 5 h, as was imposed during this FD protocol, may have been insufficient to assess light-induced sleep quality improvements during the circadian sleep phase.

Increased delta power (Figures 2 and 3) can be the consequence of both sleep deprivation, causing elevated sleep pressure levels, and increased sleep efficiency (i.e., quicker sleep pressure dissipation; Borbély et al., 1981; Williams et al., 1964). Spontaneous wake-up times could not be measured in this FD protocol; nevertheless, we argue that data presented here suggest that BL exposure increases sleep pressure buildup. Light-induced increases in sleep pressure have been determined before in both humans (Cajochen et al., 2019) and animals (Altimus et al., 2008; Hubbard et al., 2013; LeGates et al., 2014; Tsai et al., 2009). The delta power increase determined in data presented here coincided with light-induced improvements in subjective sleep quality, which occurred during the circadian wake maintenance phase (Figure 4). This suggests that BL exposure influences subjective and polysomnographic measures of sleep quality more when sleep is driven by sleep pressure as opposed to stimulated by the circadian system. Moreover, subjective sleep quality scores within the range of 0-2 indicate “undisturbed sleep,” while higher scores suggest disturbed sleep (Leppämäki et al., 2003). BL exposure may therefore restore sleep to undisturbed quality when the circadian system promotes wakefulness. Since DL light levels were lower compared to everyday light exposure levels, the discrepancy in the amount of delta power and time spent in NREM sleep between the DL and BL conditions could be the consequence of micro-sleep during DL exposure. Micro-sleep occurrence would lead to reduced sleep pressure levels and therefore relatively higher levels of delta power and more time in NREM sleep during BL exposure. Although participants were under constant surveillance during wakefulness, and other FD studies that lasted twice as long compared to our protocol did not report micro-sleep occurrence (Lazar et al., 2015), presence of these events can only be assessed by continuous EEG monitoring. It is therefore possible that BL exposure reduces micro-sleep occurrence, resulting in higher levels of sleep-wake dependent sleep pressure. Although some studies report poor correlations between polysomnographic and subjective measures (Kaplan et al., 2017, Lok et al., 2022a), sleep quality as assessed by PSG has shown to correlate to subjective sleep quality particularly when sleep is measured at alternate clock phases (O’Donnell et al., 2009), while subjective sleep quality improvements especially have been reported after (partial) sleep deprivation (Elmenhorst et al., 2008). Regardless of underlying mechanisms, subjective and polysomnographic measures presented here suggest that BL exposure may increase sleep pressure accumulation. Although this may not necessarily be advantageous for the general, healthy public that does not suffer from sleep disorders, increased sleep pressure buildup due to higher illumination exposure may help treat severe insomnia (Spielman et al., 1987) or other pathologies associated with slower buildup of sleep pressure accumulation, such as delayed sleep-wake phase syndrome (Uchiyama et al., 1999, 2000).

### Limitations

Although this was the first attempt at a successful BL FD, this study protocol comes with several limitations. First of all, financial limitations forced us to conduct this study in male participants only, which clearly hinders extrapolation to other genders. Second, the protocol duration is relatively short, encompassing merely one beat cycle. Although multiple beat cycles are desirable, a short FD design is necessary to prevent extensive light-induced tau elongations (Lok et al., 2022b, Suppl. Fig. S4), which could complicate comparison between the DL and BL conditions. Third, the chosen sleep:wakefulness ratio is 1:3, deviating from the classical 1:2 ratio. There are several reasons for this sleep-wake ratio: (1) Since uniform phase progression is essential for FD protocols, the 1:3 ratio had to be chosen, as this was the only combination that ensured equal stimulation of the phase delay and advance part of the phase response curve (Lok et al., 2022b, Suppl. Fig. S1). (2) In addition, given this protocol encompasses merely one complete beat, the 1:3 ratio ensured sleep data at all clock phases, even when the circadian system promoted wakefulness. (3) The primary goal of this study was to compare BL versus DL effects on sleep, and under both protocols, the 1:3 sleep to wakefulness ratio was used. Light effects on reported sleep measures are therefore still valid. Furthermore, simulations with the two-process model of sleep regulation show that process S does not systematically increase or decrease over the 72 h of this FD protocol (Lok et al., 2022b, Suppl. Fig. S2).

## Conclusion

In conclusion, the current study presents significant effects of BL exposure on sleep architecture, leading to sleep-pressure-related changes in sleep quality as assessed by PSG. More delta power and NREM sleep was found at the end of the scheduled sleep phase after increased light exposure, especially when sleep occurred outside the normal sleep phase but also during recovery sleep. Subjective sleep quality scores showed light-induced improvements coinciding with increased delta power and time spent in NREM sleep, suggesting that light during wakefulness may improve subsequent sleep quality. These findings may have important implications for insomnia treatment and clinical applications of light therapy.

## Supplemental Material

sj-docx-1-jbr-10.1177_07487304221096910 – Supplemental material for Bright Light During Wakefulness Improves Sleep Quality in Healthy Men: A Forced Desynchrony Study Under Dim and Bright Light (III) Click here for additional data file. Supplemental material, sj-docx-1-jbr-10.1177_07487304221096910 for Bright Light During Wakefulness Improves Sleep Quality in Healthy Men: A Forced Desynchrony Study Under Dim and Bright Light (III) by R. Lok, T. Woelders, M. C. M. Gordijn, M. J. van Koningsveld, K. Oberman, S. G. Fuhler, D. G. M. Beersma and R. A. Hut in Journal of Biological Rhythms
