# Supplementary material for: Bright Light During Wakefulness Improves Sleep Quality in Healthy Men: A Forced Desynchrony Study Under Dim and Bright Light (III)
Source: J Biol Rhythms. 2022 Jun 22;37(4):429–41. doi: 10.1177/07487304221096910 (PMC9326793; doi:10.1177/07487304221096910)
Supplement: sj-docx-1-jbr-10.1177_07487304221096910 – Supplemental material for Bright Light During Wakefulness Improves Sleep Quality in Healthy Men: A Forced Desynchrony Study Under Dim and Bright Light (III) [file sj-docx-1-jbr-10.1177_07487304221096910.docx]

**Title:** Bright light during wakefulness improves polysomnographic and subjectively assessed sleep quality in healthy men: a forced desynchrony study under dim and bright light (III).

**Running title:** Bright light during wakefulness improves sleep quality.

**Authors:** R. Lok^1,2,3,^*, T. Woelders^1,3^, M.C.M. Gordijn ^1,4^ , M.J. van Koningsveld^1^, K. Oberman^1^, S.G. Fuhler^1^, D.G.M. Beersma^1^, R.A. Hut^1^

**Contact Information: ^1^**University of Groningen, Chronobiology unit, Groningen Institute for Evolutionary Life Sciences, PO box 11103, 9700CC, Groningen, the Netherlands.

**^2^**University of Groningen, Campus Fryslân, Wirdumerdijk 34, 8911 CE, Leeuwarden, the Netherlands.

^3^ Both authors contributed equally

^4^Chrono@Work B.V., Friesestraatweg 213, 9743 AD, Groningen, The Netherlands

^*^ To whom all correspondence should be addressed: Renske Lok, University of Groningen, Chronobiology Unit, Present address: Department of Psychiatry and Behavioral Sciences, Stanford University, 401 Quarry Road, Palo Alto, CA, 94305, [rlok@stanford.edu](mailto:rlok@stanford.edu)

**Supplemental information.**


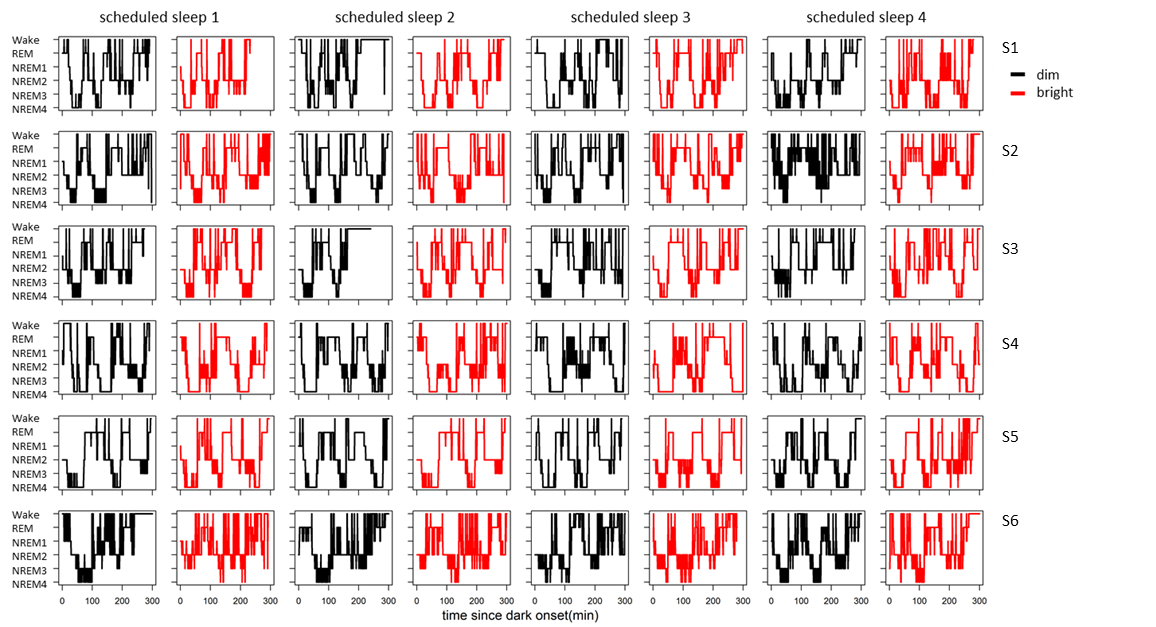


**Figure S1: Individual hypnograms.** States of wakefulness, REM and non-REM stages are indicated from top to bottom. Every row represents an individual (S1-S6) during all four scheduled sleep opportunities in the dim (black) and bright light (red) condition.


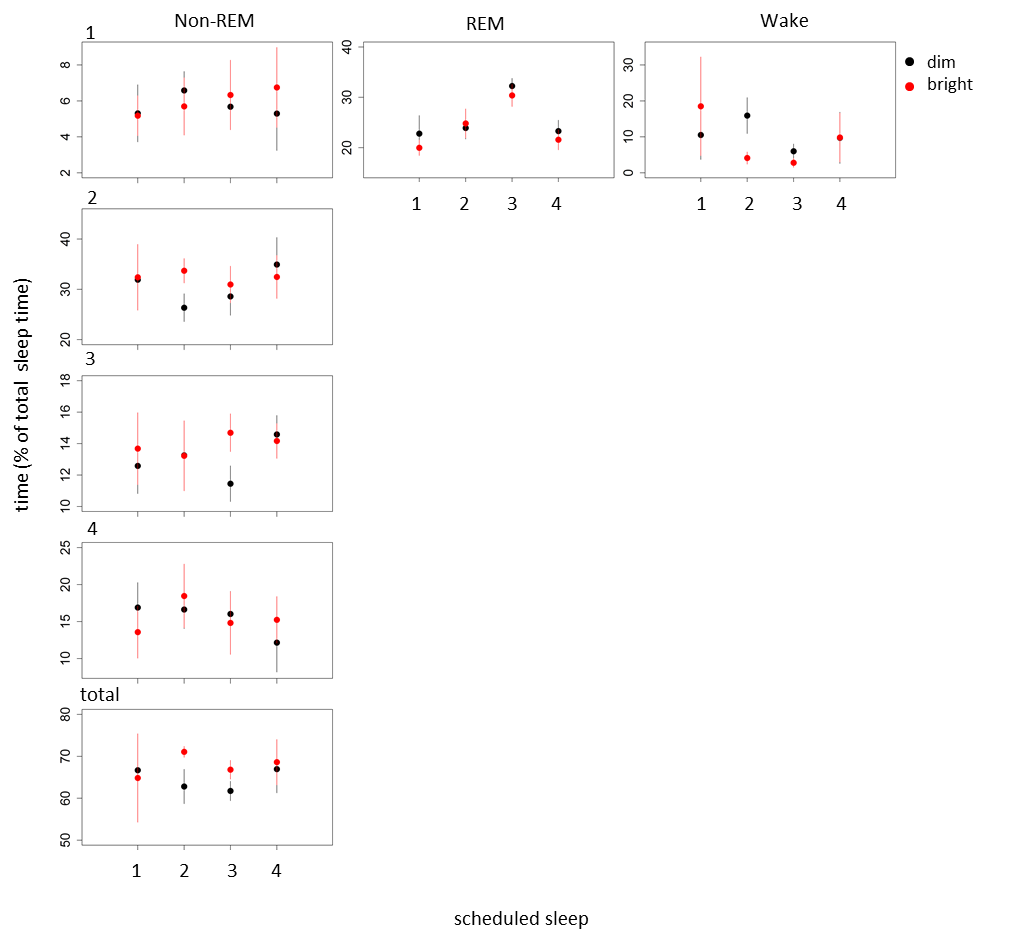


**Figure S2: Amount of time spent in Non-REM stage 1-4, summation of non-REM stages 1-4, REM and wakefulness.** Dim and bright light are indicated in black and red respectively, with n=6 individuals.

**Table S1: Quantification of time spent (units = minutes) in non-REM sleep, REM sleep and wakefulness during each scheduled sleep episode.** Data represent n=6 individuals.

|  |  | Non-REM | REM | Wake |
| --- | --- | --- | --- | --- |
| Scheduled sleep 1 | D | 66.70 ± 4.58 | 22.78 ± 3.61 | 10.52 ± 6.84 |
|  | B | 64.84 ± 10.57 | 19.98 ± 1.55 | 18.51 ± 13.76 |
| Scheduled sleep 2 | D | 62.81 ± 4.13 | 23.91 ± 2.20 | 15.93 ± 5.03 |
|  | B | 71.07 ± 1.31 | 24.81 ± 2.90 | 4.11 ± 1.71 |
| Scheduled sleep 3 | D | 61.74 ± 2.34 | 32.26 ± 1.53 | 5.99 ± 2.05 |
|  | B | 66.81 ± 2.25 | 30.39 ± 2.22 | 2.79 ± 1.30 |
| Scheduled sleep 4 | D | 66.96 ± 5.69 | 23.30 ± 2.18 | 9.73 ± 7.15 |
|  | B | 68.62 ± 5.43 | 21.57 ± 2.01 | 9.81 ± 6.82 |


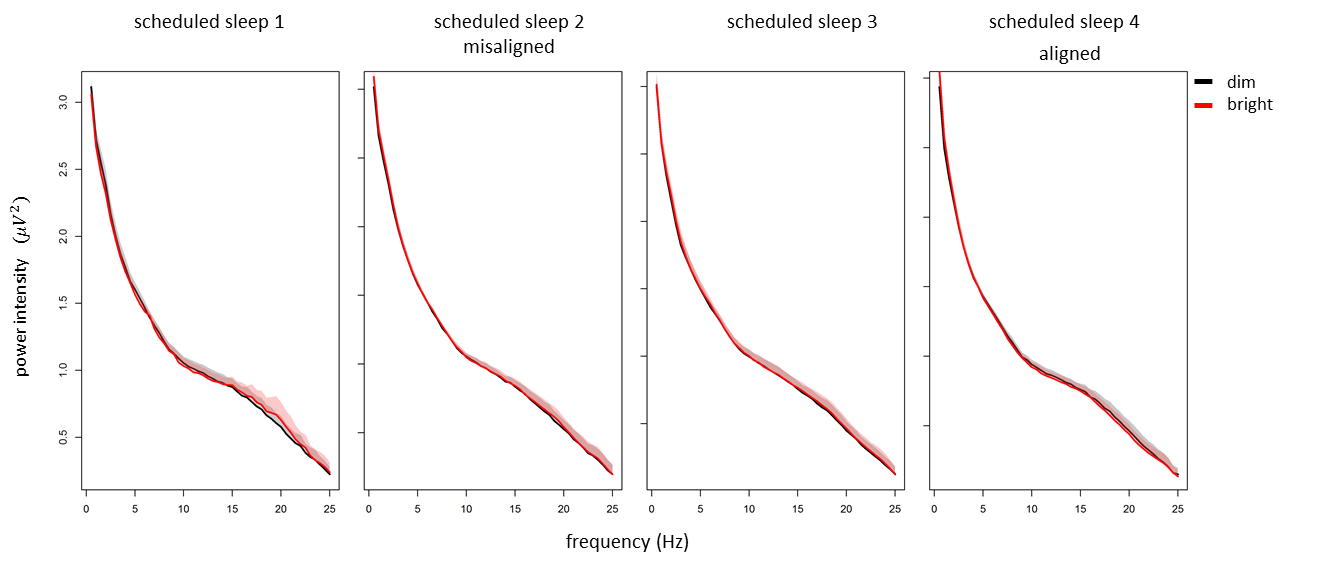


**Figure S3: Spectrograms of scheduled sleep 1 - 4.** Dim and bright light are indicated in black and red respectively, with n=6 individuals. No significant differences between light conditions were detected.


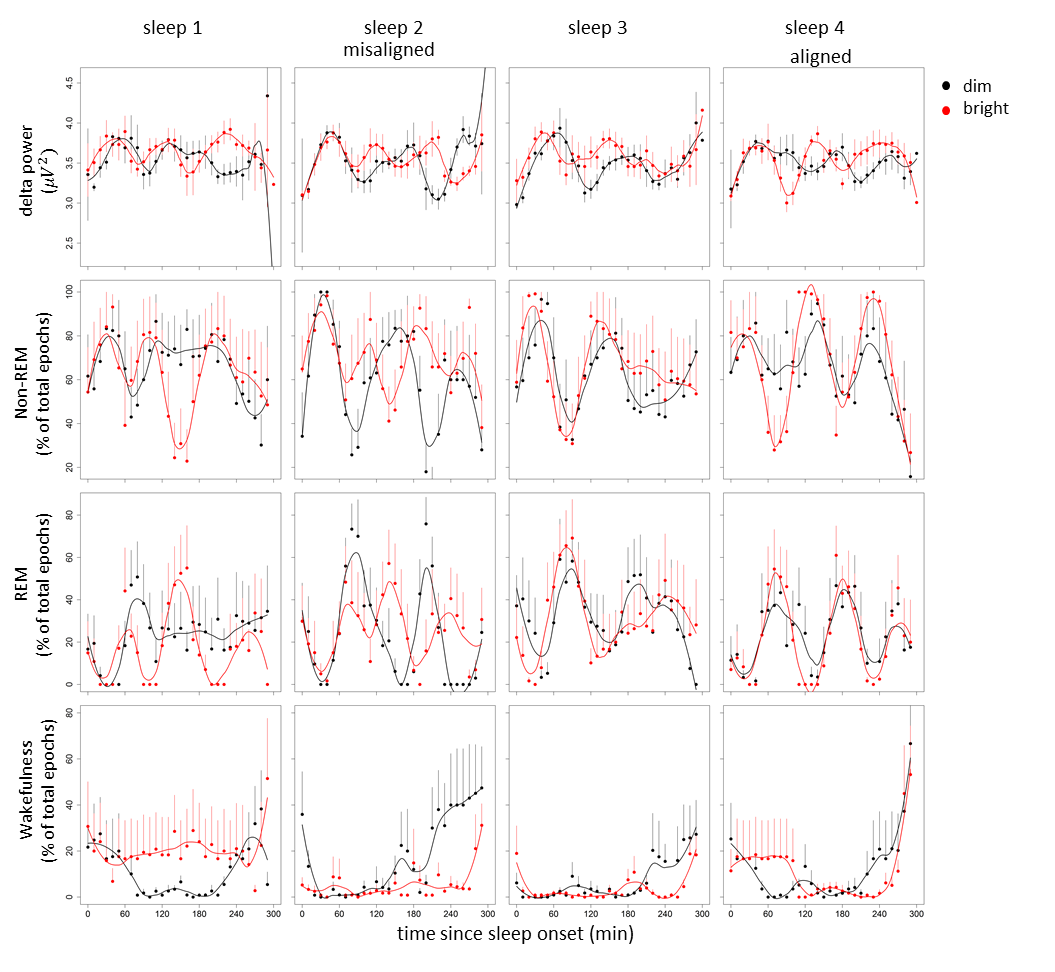
**Figure S4. The amount of Delta power, time in NREM, REM and wakefulness as a percentage of total sleep time per sleep opportunity.** Dim and bright light are depicted in black and red respectively.


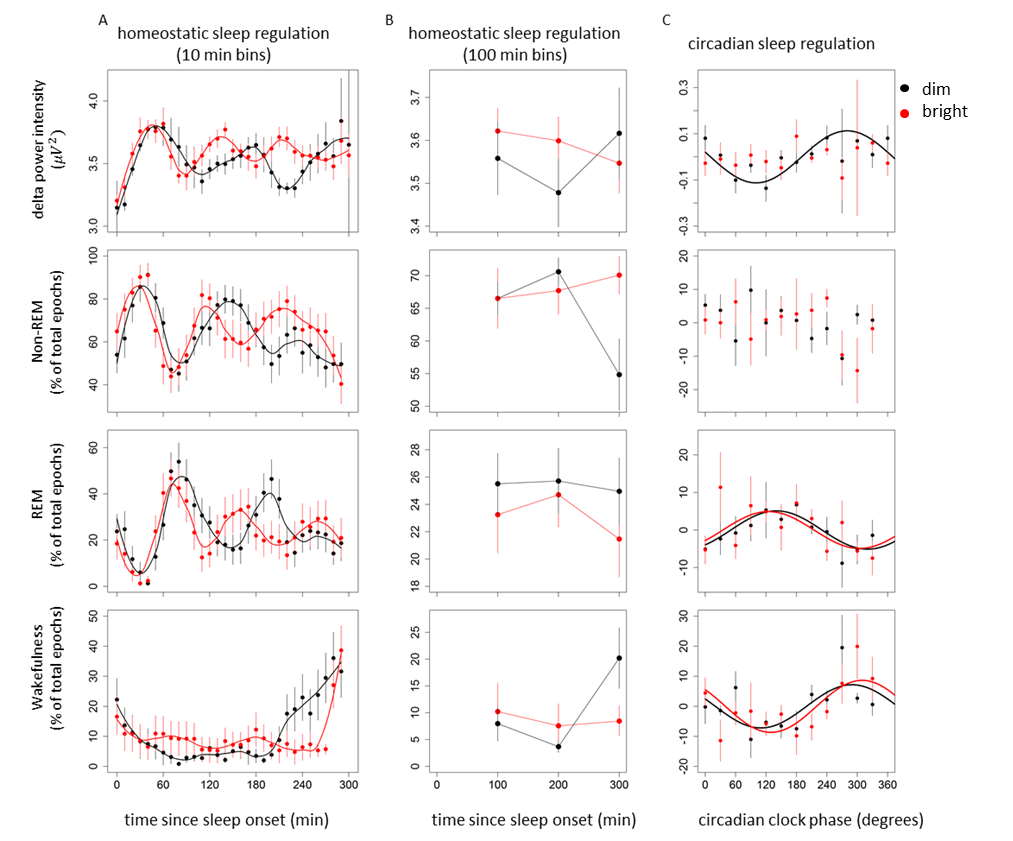


**Figure S5. The amount of NREM power density, time in NREM, REM and wakefulness plotted against time since sleep onset in (A) 10 minutes bins, (B) 100 minute bins, and (C) versus internal clock time.** Dim and bright light are depicted in black and red respectively, with n=6 individuals per light condition.


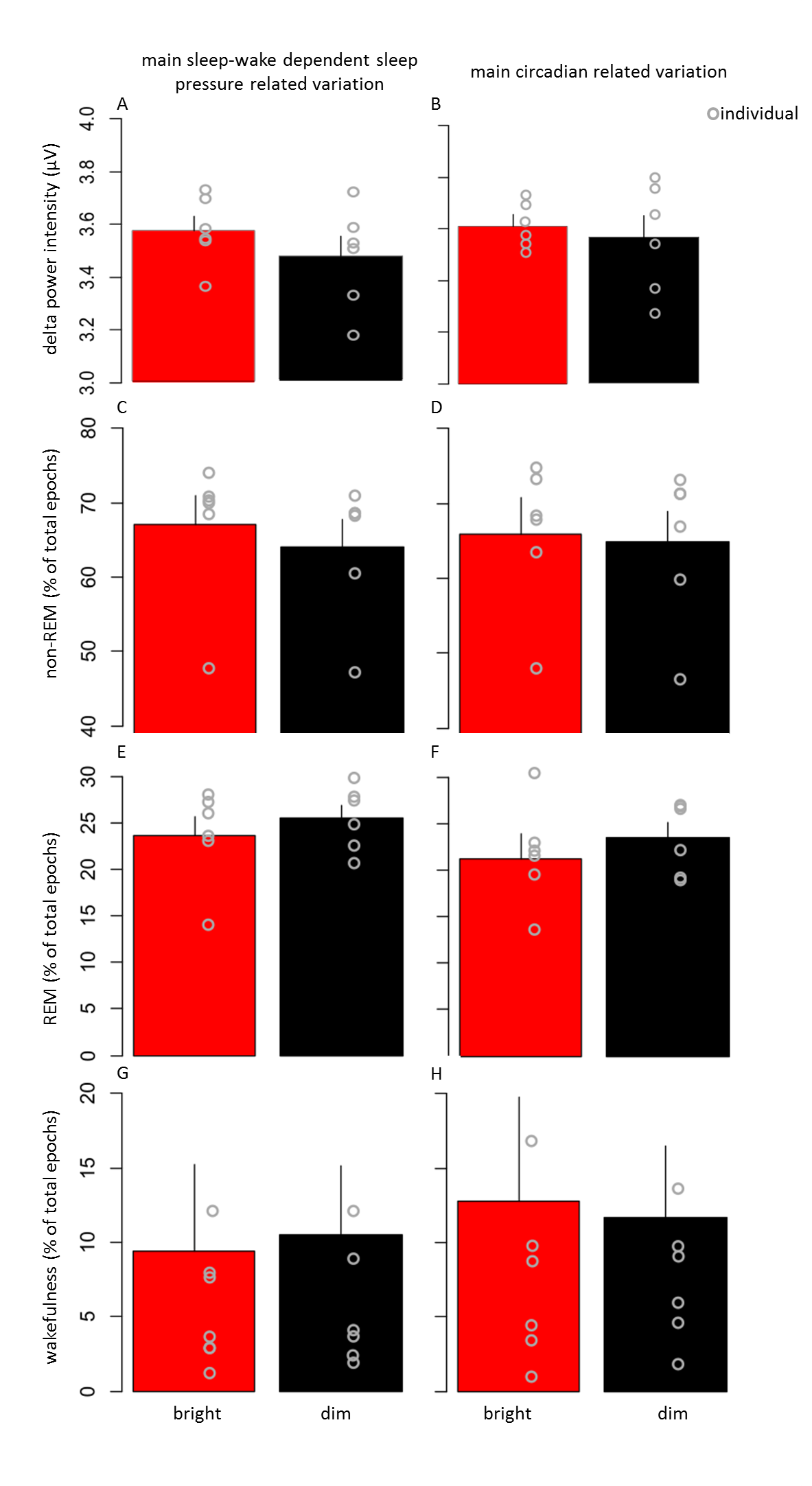


**Figure S6. The amount of NREM power density, time in NREM, REM and wakefulness plotted against time since sleep onset in (A) 10 minutes bins, (B) 100 minute bins, and (C) versus internal clock time.** Dim and bright light are depicted in black and red respectively, with n=6 individuals per light condition.

**Table S2: Statistics of sleep power accumulation in Delta power, time spent in NREM, REM and wakefulness per scheduled sleep and during recovery sleep.**

|  | *Scheduled sleep* | Light | *Df* | χ2 | *p* |
| --- | --- | --- | --- | --- | --- |
| Delta power | 2 |  | 1 | 0.11 | 0.81 |
|  | 2 | interaction | 20 | 17.72 | 0.61 |
|  | 3 |  | 1 | **5.35** | **0.02** |
|  | 3 | interaction | 20 | **194.18** | **2.2e-16** |
|  | 4 |  | 1 | 3.56 | 0.06 |
|  | 4 | interaction | 20 | 64.47 | 2.93 |
|  | 5 |  | 1 | 0.45 | 0.50 |
|  | 5 | interaction | 20 | 26.95 | 0.14 |
| NREM | 2 |  | 1 | 0.09 | 0.77 |
|  | 2 | interaction | 20 | **41.38** | **3.0e-3** |
|  | 3 |  | 1 | 3.65 | 0.06 |
|  | 3 | interaction | 20 | 115.47 | 0.08 |
|  | 4 |  | 1 | **2.97** | **0.01** |
|  | 4 | interaction | 20 | 57.06 | 0.46 |
|  | 5 |  | 1 | 0.53 | 0.46 |
|  | 5 | interaction | 20 | **55.32** | **4.0e-3** |
| REM | 2 |  | 1 | 0.54 | 0.46 |
|  | 2 | interaction | 20 | **51.5** | **1.0e-4** |
|  | 3 |  | 1 | 0.13 | 0.72 |
|  | 3 | interaction | 20 | 29.24 | 0.083 |
|  | 4 |  | 1 | 0.51 | 0.47 |
|  | 4 | interaction | 20 | 24.94 | 0.20 |
|  | 5 |  | 1 | 0.95 | 0.33 |
|  | 5 | interaction | 20 | 28.41 | 0.10 |
| Wake | 2 |  | 1 | 1.32 | 0.25 |
|  | 2 | interaction | 20 | **51.13** | **1.5e-4** |
|  | 3 |  | 1 | **5.01** | **0.025** |
|  | 3 | interaction | 20 | **266.54** | **2.2e-16** |
|  | 4 |  | 1 | **4.64** | **0.03** |
|  | 4 | interaction | 20 | **205.88** | **2.2e-16** |
|  | 5 |  | 1 | 0.0019 | 0.96 |
|  | 5 | interaction | 20 | 26.13 | 0.16 |
| Power | Recovery |  | 1 | **4.99** | **0.025** |
| Power | Recovery | interaction | 20 | **262.24** | **2.2e-16** |
| NREM | Recovery |  | 1 | **85.04** | **0.023** |
| NREM | Recovery | interaction | 20 | **254.66** | **2.2e-16** |
| REM | Recovery |  | 1 | 1.39 | 0.24 |
| REM | Recovery | interaction | 20 | 30.74 | 0.059 |
| Wake | Recovery |  | 1 | 1.46 | 0.23 |
| Wake | Recovery | interaction | 20 | **72.13** | **8.16e-8** |

**Light effects on sleep architecture and -quality during recovery sleep.**

*Sleep-wake dependent sleep pressure variation (process S):* At the end of the FD paradigm, there was a 2 min obligatory awakening to fill in the GSQS questionnaire, where after participants were requested to enter sleep again. This recovery sleep lasted for a maximum of 3 hours, and all participants used this opportunity for sleep. Measurements of that period of sleep suggest a decrease in the amount of time spent in NREM sleep over time (Fig 5C), while the amount of time in REM sleep increased (Fig 5E).

*Sleep accumulation:* Cumulative scores indicate more accumulated delta power after bright light exposure (Fig 5B) as well as time spent in NREM sleep (Fig 5D, Table S2). This was (partially) at the expense of wakefulness, which decreased after bright light exposure (Fig 5H, Table S2), while REM accumulation was unaffected (Fig 5F). Recovery sleep offset times did not differ between both light conditions (Fig S7).


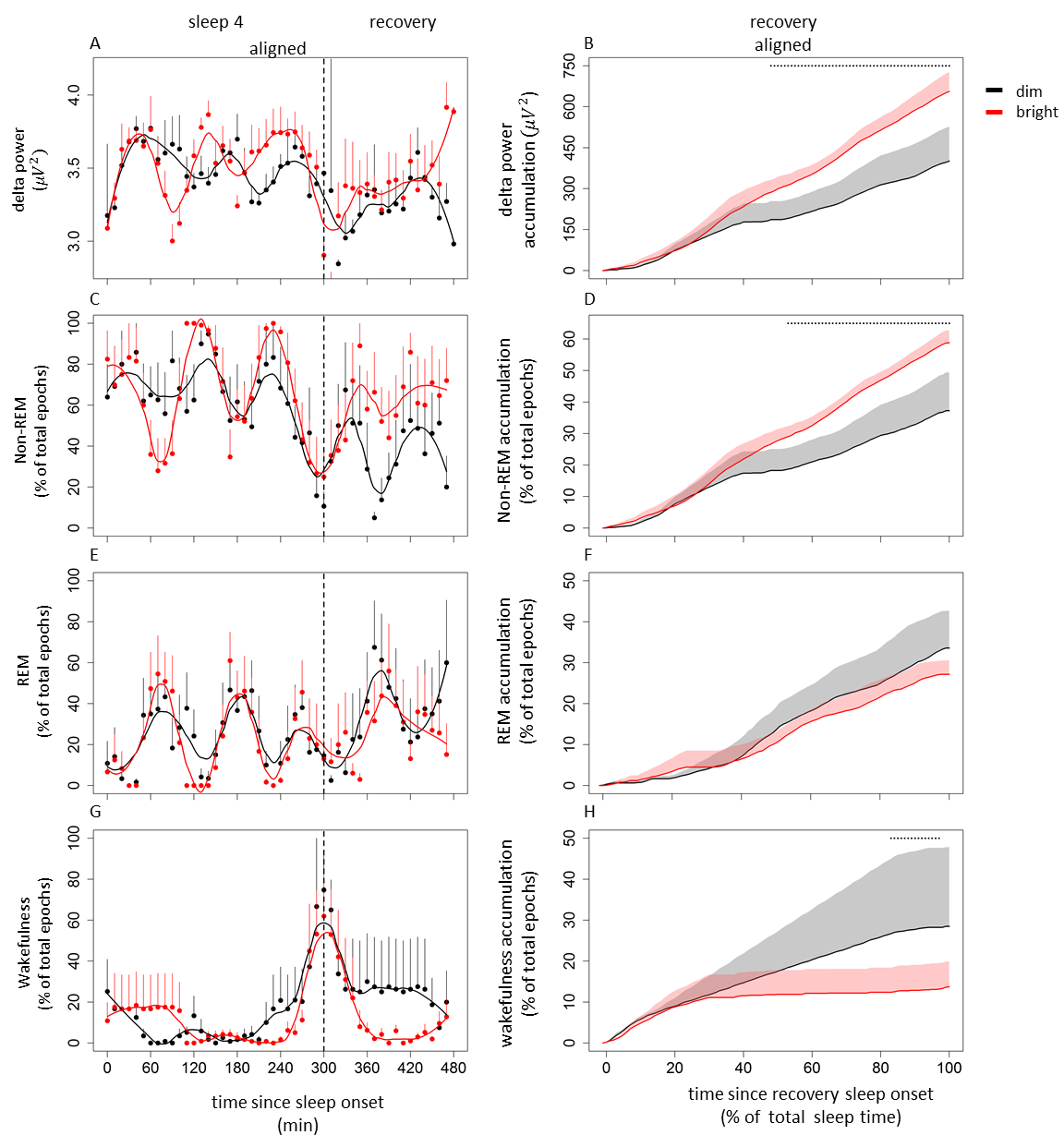


**Figure S7. Bright light induced recovery sleep changes (N=6).** Amount of delta power (A-B), time in NREM sleep (C-D), REM sleep (E-F) and wakefulness (G-H) as a percentage of total sleep time and accumulated over the recovery sleep window for the dim (black) and bright (red) light condition. The dashed line (A, C, E, G) indicates obligatory awakening, followed by the 3-h sleep opportunity that provided the data for the cumulative plots (B, D, F, H). Dotted horizontal lines indicate significant differences between light conditions.


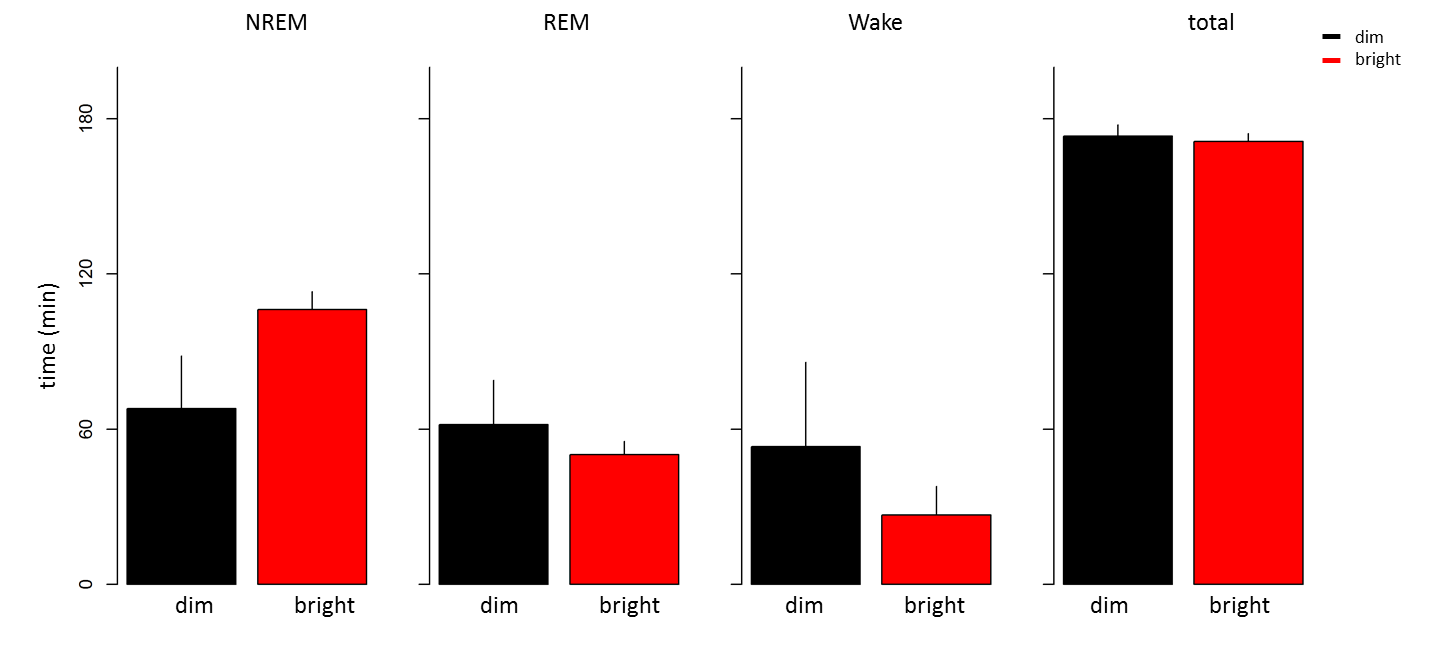


**Figure S8: Average (± SEM) time spent in NREM-, REM sleep and wakefulness and total sleep time during recovery sleep.** Dim and bright light are indicated in black and red respectively, with n=4 individuals. After bright light exposure, there was significantly more time spent in NREM sleep, at the cost of wakefulness. Nonetheless, there were no significant differences in total sleep time between conditions.
